# A Wireless ExG Interface for Patch-Type ECG Holter and EMG-Controlled Robot Hand

**DOI:** 10.3390/s17081888

**Published:** 2017-08-16

**Authors:** Kwangmuk Lee, Yun Young Choi, Dae Jung Kim, Hee Young Chae, Kyeonghwan Park, Young Min Oh, Sung Hun Woo, Jae Joon Kim

**Affiliations:** 1 School of Electrical and Computer Engineering, Ulsan National Institute of Science and Technology, Ulsan 44919, Korea; leekm0226@unist.ac.kr (K.L.); laplume@unist.ac.kr (H.Y.C.); khpark@unist.ac.kr (K.P.); 2 UMEDIX Co., Ltd., Seoul 06097, Korea; yychoi.choi@gmail.com (Y.Y.C.); djnara@gmail.com (D.J.K.); oym.hazin@gmail.com (Y.M.O.); steven.shucom.woo@gmail.com (S.H.W.)

**Keywords:** motion artifact, readout integrated circuit, ECG holter, robot-hand controller, envelope detector, level detector

## Abstract

This paper presents a wearable electrophysiological interface with enhanced immunity to motion artifacts. Anti-artifact schemes, including a patch-type modular structure and real-time automatic level adjustment, are proposed and verified in two wireless system prototypes of a patch-type electrocardiogram (ECG) module and an electromyogram (EMG)-based robot-hand controller. Their common ExG readout integrated circuit (ROIC), which is reconfigurable for multiple physiological interfaces, is designed and fabricated in a 0.18 μm CMOS process. Moreover, analog pre-processing structures based on envelope detection are integrated with one another to mitigate signal processing burdens in the digital domain effectively.

## 1. Introduction

Electrophysiological monitoring devices such as electrocardiograms (ECGs) and electromyograms (EMGs) have inherent artifacts; that is, anomalous or interfering signals that originate from some sources other than the electrophysiological structure. Especially in wearable-type devices, which are preferred for 24-h monitoring systems and prosthesis control devices [1,2], the motion artifact is a crucial factor that dominates the overall monitoring quality, because most physiological devices generally operate in direct contact with human bodies [3,4]. Various physical movements give unstable skin contact, causing variations in the contact impedance and resulting in transient signal fluctuations. However, these motion artifacts are not easy to eliminate, because their frequency band overlaps with the frequency bands of electrophysiological signals, which degrades accuracy in the physiological signal analysis [5]. Therefore, there have been recent works to alleviate this motion-artifact problem by utilizing adaptive filtering [6], baseline wander tracking [7] or bio-impedance measurement [8]. However, the filtering works require additional detection channels for a reference, and baseline tracking requires a complex control loop that consists of an analog-to-digital converter (ADC), a digital-to-analog converter (DAC), and digital signal processing (DSP). The bio-impedance measurement is dedicated to heart rate, which is not appropriate for multi-sensor purposes. In terms of low-power DSP implementation, recent digital-domain efforts such as down-sampled wavelet transform [9], sub-Nyquist scanning [10], and data compression [11] have been reported to reduce the data rate from sensor modules or to relieve their post-processing and data-storage burdens. But, these digital efforts are still limited, because they do not fundamentally reduce the data rate of the sensors themselves. Meanwhile, one interesting analog pre-processing work on R-wave timing extraction from ECG measurement [12], whose digital implementation consumes three times more than its analog front end, has been reported. Even though it was implemented only for heart-rate detection, it is meaningful that analog pre-processing can reduce the burden of its digital-domain post-processing work. Therefore, conceptually inspired by this analog pre-processing method, we propose a reconfigurable ExG interface structure that is tolerant of various motion artifacts, and also reduces its post-processing burden in digital domain efficiently.

This work presents two wireless system prototypes of a patch-type ECG holter and an EMG-based robot-hand controller and their common ExG readout integrated circuit (ROIC) with multiple proposed circuit- and system-level technologies for motion-artifact reduction. In the case of the ECG detection, which has stringent requirements in terms of signal quality, its sensing module is made flexible by implementing each of its components on flexible principle printed circuit boards (PCBs); then, the whole module, including electrodes, is immobilized by putting adhesive patches on it. Through this patch-type implementation, a large portion of the motion artifacts are supposed to be autonomously removed. Then, in order to minimize the remaining artifacts, instant amplitude fluctuations are automatically compensated for by a proposed level detection scheme, and are also minimized by adopting state-of-the-art methods such as impedance boosting [13] and chopper stabilization [14]. 

For the EMG-based robot-hand control, the EMG module, whose signal-quality requirement is less strict, is designed in portable form for durability and reusability. Since the EMG signal has a higher frequency band, an envelope detection method is proposed to relieve its digital post-processing burden. Similarly, the R-peak timing information that is required for the ECG signal analysis in the digital domain is provided by a proposed simple analog method of a peak detection, resulting in significant reduction of its post-processing loads. For feasibility verification of these proposed methods, an ExG ROIC prototype and two system-level prototypes are fabricated and experimentally verified. 

The remainder of this paper is organized as follows: Section 2 presents the proposed ExG interface architectures. Section 3 contains detailed designs of the ExG ROIC. Section 4 shows experimental results. Finally, the conclusion is given in Section 5.

## 2. ExG Interface Architecture

**Figure 1:**
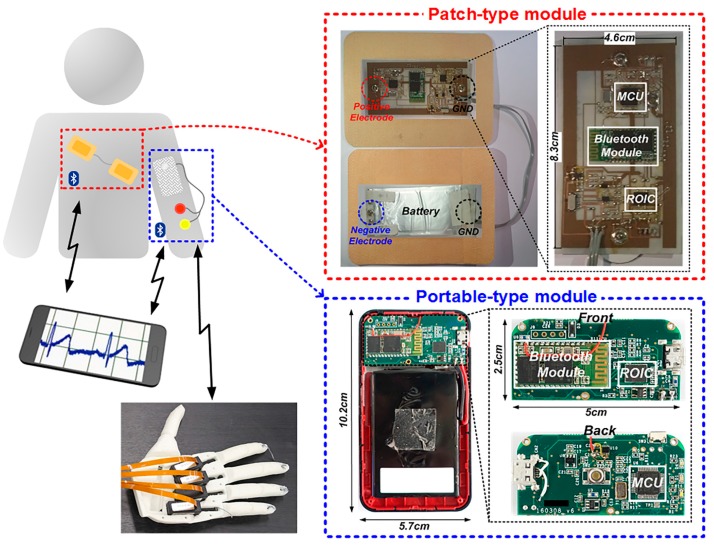
Wireless ExG interface with patch-type and portable-type modules for mobile healthcare and robot-hand control applications.

Figure 1 shows a proposed wireless ExG interface system to support the ECG monitoring and the EMG-based control, where wireless connectivity and battery are included together to minimize potential motion artifacts paths. The overall system is embodied as two wireless modules of a patch-type ECG holter and a portable-type EMG-based robot-hand, where a designed ExG ROIC is commonly utilized and their monitoring results are remotely displayed on a smartphone. For stable ambulatory ECG monitoring and effective reduction of motion artifact, a patch-type module is designed to implement most components on a flexible PCB that has polydimethylsiloxane (PDMS) substrate. One side of the module consists of the ExG ROIC, a microprocessor (MCU), a Bluetooth module, and two electrodes of the positive and the ground. The other side has a flexible lithium-ion battery and two electrodes of the negative and the ground. Through this hardware structure, two electrodes of the positive and the negative are autonomously located to have proper interspace around the heart for the ECG signal detection. For user convenience to control the robot hand intuitively, the EMG interface is designed to be a portable-type module which has similar composition of hardware components in the patch-type. The size of core PCB board is 5 × 2.5 cm which is smaller than the ECG module and the rest area is filled with battery to give enough operation time. The EMG interface is configured to detect EMG signals from the arm muscles and to control the robot hand [15] wirelessly.

**Figure 2:**
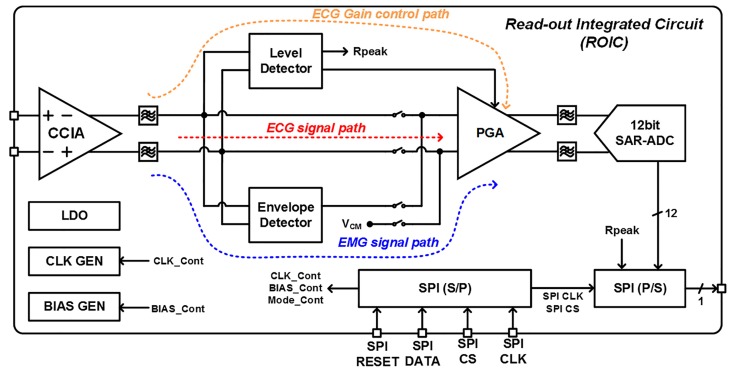
Block diagram of ExG readout integrated circuit (ROIC) for ECG/EMG interfaces.

Figure 2 shows the architecture of the ExG ROIC that is commonly used in the two application systems. It consists of a capacitive-coupled instrumentation amplifier (CCIA), a programmable-gain amplifier (PGA), a level detector for real-time automatic gain calibration, and an envelope detector for amplitude detection of EMG signals. A low dropout (LDO) regulator, clock generators, bias circuits, a 12-bit successive approximation register (SAR) ADC [16], and a serial peripheral interface (SPI) are integrated together which includes a serial-to-parallel (S/P) register and parallel-to-serial (P/S) register. It is also designed to have reconfigurable structure to adjust voltage gain and frequency band depending on bio-potential signal types. In the ECG signal path, the PGA gain is automatically adjusted by a proposed level detector. The EMG path has another signal processing procedure of the envelope extraction to reduce its frequency bandwidth, which relieves its post-processing burden in digital domain. In this way, the proposed ROIC provides reconfigurable ExG interface whose internal operation mode is software-controlled. Another benefit of this software-controlled reconfigurable ROIC is to provide excellent protection capability from the reverse engineering of integrated circuits or systems because the hardware operation cannot be understood without its software information.

## 3. ROIC Implementation

### 3.1. CCIA and PGA

**Figure 3:**
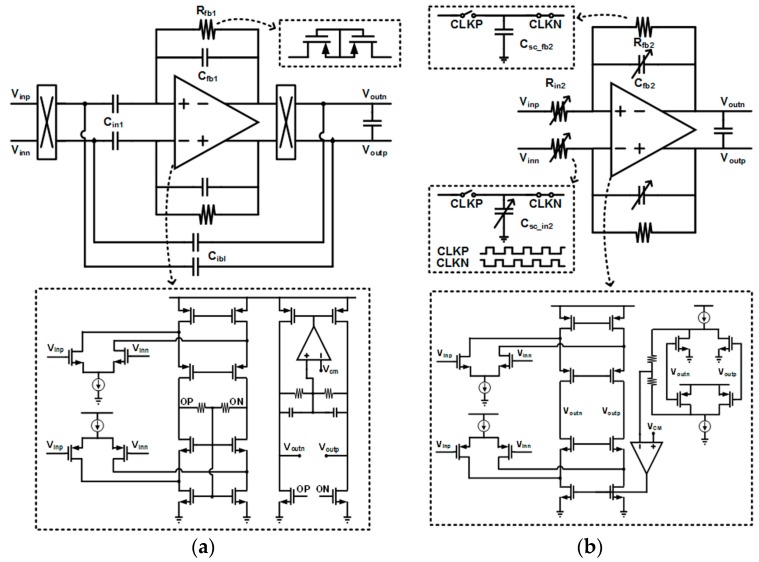
Schematics of (**a**) CCIA and (**b**) PGA.

The schematic of the CCIA, which is a front-end low-noise amplifier to remove low-frequency noise and DC offsets, is shown in Figure 3a, where it employs capacitive feedback to set mid-band gain of C_in1_/C_fb1_. The input capacitor C_in1_ and the feedback capacitor C_fb1_ are designed to be 5 pF and 250 fF, respectively, in order to realize the 26 dB mid-band gain. The chopper stabilization technique [14] is adopted to suppress low-frequency noises including 1/f noises. The input impedance is increased further to minimize remaining motion-artifact effects by adopting capacitive impedance boosting [13], which implements a positive feedback between input and output nodes without additional current consumption. The resulting input impedance is maximized when C_ibl_ is almost equal to C_fb1_. In order to achieve a low high-pass cutoff frequency for DC-offset reduction, the feedback resistor R_fb1_ is implemented with a PMOS pseudo-resistor that achieves very high resistance in small area [17].

The schematic of PGA in the second stage, which is a kind of switched-capacitor amplifier, is shown in Figure 3b, where the variable resistors R_in2_ and R_fb2_ are implemented by capacitors of C_sc_in2_ and C_sc_fb2_, and switches with non-overlapping clocks of CLKP and CLKN. The PGA is designed to set mid-band gain (C_sc_in2_/C_sc_fb2_), and its low-pass cutoff frequency is changed by adjusting the feedback capacitor C_fb2_ based on the operation modes of the ECG and the EMG. For sufficient noise immunity, fully-differential amplifiers (FDAs) in the PGA and the CCIA are commonly designed to have folded-cascode structures with rail-to-rail input ranges. While the FDA for the CCIA, including its common-mode feedback circuits, is optimized to minimize the power consumption, the FDA for the PGA is designed to have current-driving capability for output loads.

### 3.2. Envelope and Level Detectors

**Figure 4:**
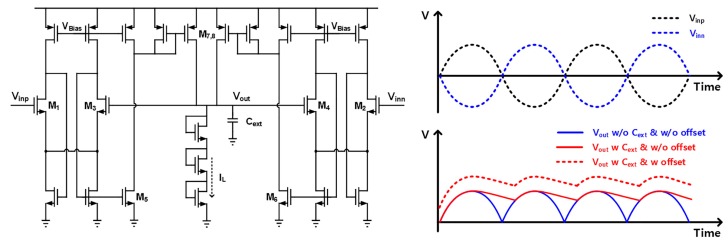
Schematic and operational principle of the envelope detector.

The robot-hand system is designed to be controlled by referring the EMG signal strength, where important control information exists in the amplitude of instantaneous EMG spikes. Therefore, the EMG signals are converted to low-frequency amplitude signals by utilizing the envelope detector, reducing their sampling rate in the process of digital conversion, and thereby reducing the digital post-processing burden considerably. The schematic and basic operation of the designed envelope detector, which is based on the peak detector in [18], are shown in Figure 4. If any input among V_inp_ and V_inn_ is greater than the output of V_out_, the current mirror chain of M_5,6_ and M_7,8_ provides current proportional to their difference, and the peak value of the inputs is stored in the output through an external capacitor C_ext_. For continuous tracking of time-varying input signals, the output voltage is slowly discharged by utilizing a leakage current I_L_, which comes from three diode-connected transistors. Since the size ratio of M_1,2_ and M_3,4_ affects the feedback amount, the output DC offset can be adjusted by changing the M_3,4_ size.

**Figure 5:**
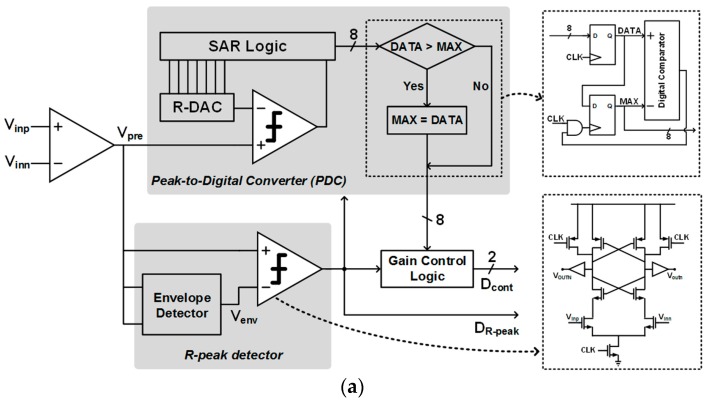

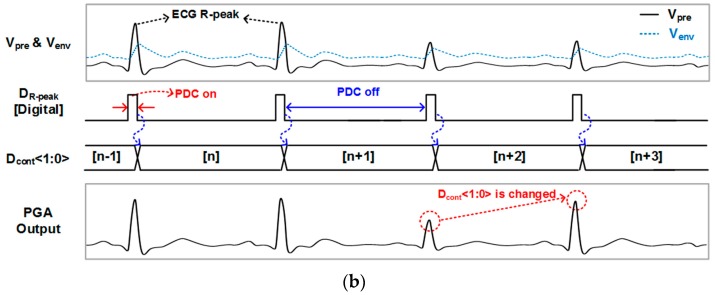
ECG level detector: (**a**) circuit diagram and (**b**) operation principle.

Figure 5 shows the architecture of the proposed ECG level detector and the operational principle of its automatic gain control on the PGA with detection capability of the R-peak signal. The envelope detector is used to find the R-peak in analog domain to reduce the post-processing burden that finds the R-peak location of the ECG signal in digital domain, where it is designed to have some output offset-voltage control range by adjusting the size of M_3,4_. The input signal of the level detector, which is processed to V_pre_, comes from a differential output of CCIA, which is a first-stage amplifier in the ROIC. The envelope detector generates the ECG baseline signal with some offset (V_env_), and it is compared with V_pre_ to produce the R-peak location signal of D_R-peak_. If the D_R-peak_ becomes too high, it activates a peak-to-digital converter (PDC), which is composed of a comparator, an 8-bit resistive digital-to-analog converter (DAC), and successive approximate register (SAR) control logic. The PDC output, which represents a digital value of the R-peak signal in the ECG, is converted to the PGA control signal of D_cont_. In this way, the ECG signal amplitude is automatically controlled to give stable output waveforms, making the overall system less susceptible to motion-artifact fluctuations. The sensitivity of this automatic amplitude control can be adaptively changed by tuning the programming of the mapping method from D_R-peak_ to D_cont_, depending on operating environments.

## 4. Experimental Results

**Figure 6:**
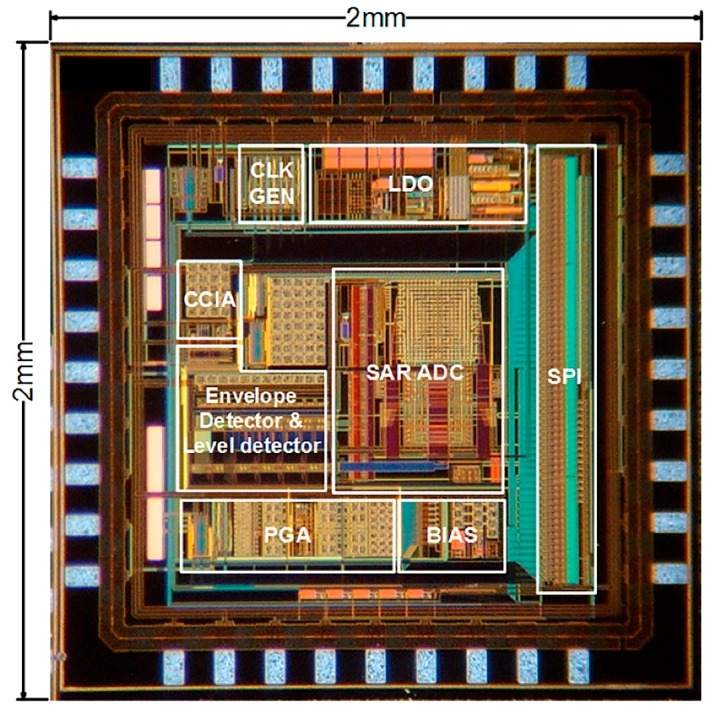
Microphograph of ExG ROIC prototype.

**Figure 7:**
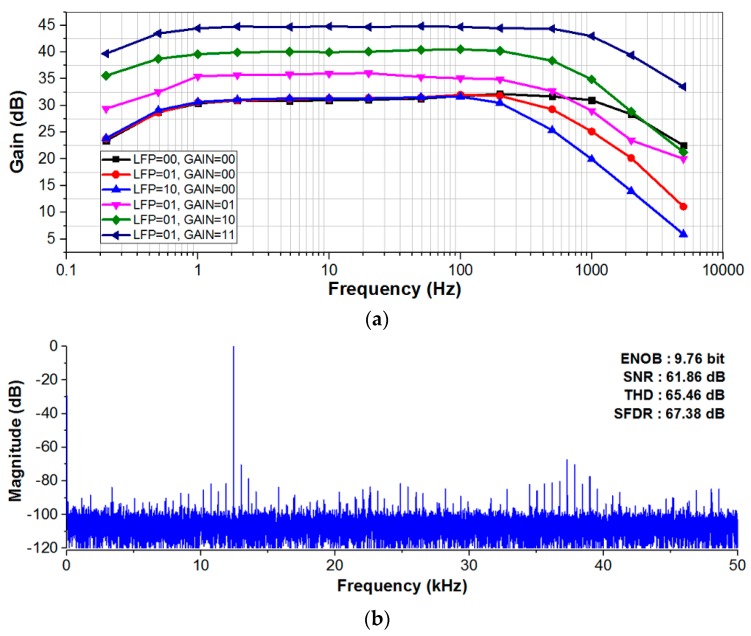
Measured characteristics of (**a**) ExG ROIC; and (**b**) SAR ADC.

A proposed ExG ROIC prototype was fabricated in a 0.18 μm complementary metal oxide semiconductor (CMOS) process, and Figure 6 shows its microphotograph with a chip area of 4 mm^2^. The external supply voltage is 3 V, but the LDO inside the ROIC provides an internal supply voltage of 1.8 V for better noise immunity and device protection. For verification of the ROIC prototype, the gain control capability was measured as shown in Figure 7a, where the low-pass corner frequency of the PGA was adjusted together. The measured pass band was set to be 0.6 to 230 Hz in the ECG mode and 0.6 to 1.5 kHz in the EMG mode. The measured programmable pass-band gain range was 31.3 to 44.8 dB, and the power consumption was 37.3 μW. At 60 Hz, the common-mode rejection ratio (CMRR) was 65.6 dB and power-supply rejection ratio (PSRR) was 55.4 dB. Figure 7b shows measured characteristic of the SAR ADC, where the spurious-free dynamic range (SFDR) is 67.38 dB and the power consumption is 0.2 μW at 125 S/s sampling rate.

**Figure 8:**
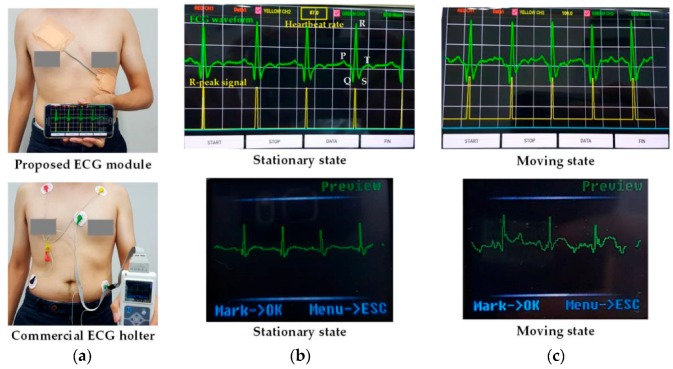
Comparison of experimental results of the proposed ECG prototype versus commercial ECG holter (TLC5000 of Contec Medical Systems). (**a**) Experiment environment; (**b**) Comparison results in stationary state; (**c**) Comparison results in moving state.

By utilizing the fabricated ExG ROIC, two system-level prototypes of the patch type and the portable type were manufactured as seen in Figure 1. First, the ECG module that consists of the ROIC, electrodes (3 M Ag/AgCl), the MCU (ARM cortex-M0), the Bluetooth (HC-06), and the flexible battery was implemented as patch-type to immobilize every component for anti-artifacts. For feasibility verifications of the proposed motion-artifact reduction methods, the comparison experiment of the wireless ECG patch prototype against a commercial ECG holter product (TLC5000 of Contec Medical Systems) was performed as shown in Figure 8, where electrodes are attached according to the standard 12-lead ECG placement [5]. The commercial holter’s waveform corrupted seriously when the human moved up and down (in moving state), while the proposed prototype gave stable waveforms in both stationary and moving states, and the P-QRS-T wave was clearly observed. In the proposed prototype, the R-peak waveform, which is generated from the proposed level detector, was displayed together, which would initiate its digital post-processing sequences to analyze the ECG waveform characteristics. Through this comparison experiment, it can be seen that the proposed anti-artifact methods are more attractive in the form of wearable devices because the adhesive patch increases the anti-artifact capability of the ROIC.

**Figure 9:**
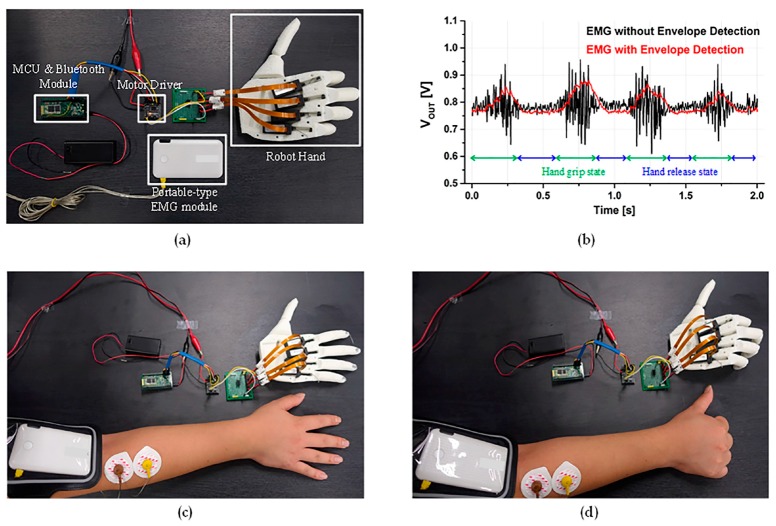
Experiment of robot-hand controller interface. (**a**) Experiment environment; (**b**) EMG waveform with and without envelope detection; (**c**) Robot-hand control in hand release state; (**d**) Robot-hand control in hand grip state.

Figure 9a shows the experimental environment of EMG-based wireless robot-hand control interface, which is composed of the proposed portable-type EMG module and a robot-hand controller. The robot-hand controller consists of the MCU, the Bluetooth, a DC-motor driver, and a commercial robot hand of Open Bionics, which includes one micro linear actuator at each finger. During this experiment, the EMG envelope waveform depending hand movement is shown in Figure 9b. The EMG envelope signal shows low-frequency amplitude waveforms, while the original EMG signal gives high-frequency AC waveforms. The robot-hand control interface for mimicking hand activity is easily implemented by utilizing the EMG envelope signal, rather than the original. In Figure 9c,d, it can been seen that the robot fingers moved following the human finger movement intuitively. Through this experiment, the effectiveness of the envelope-based EMG interface to simplify its post-processing was verified, where intuitive control capability of the robot hand was achieved through the EMG-based mimic mechanism. 

In this way, the designed common ExG interface was experimentally verified to support both the ECG and the EMG, which provides a one-channel sensor interface. Measured performances are summarized in Table 1, where comparisons with recent works are also given. The designed ROIC provides multiple electrophysiological interfaces through the proposed reconfigurable structure, embedding distinguished features of anti-artifact and pre-processing functions. For further applications, including standard 12-lead ECG recording [5] and multi-point EMG detection of various muscle movements [15], the common ROIC would be redesigned to support multi-channel ExG interfaces, where Bluetooth might need to be replaced with WiFi in order to cover multiple streams of data traffic from multiple channels. 

## 5. Conclusions

A reconfigurable ExG interface structure that is immune to motion artifacts and reduce digital post-processing burden considerably was proposed and experimentally verified in the form of two wireless electrophysiological system prototypes. For ECG interface, the patch-type wireless sensing module was developed to include two proposed features of automatic level adjustment and R-peak timing pre-processing. The wireless robot-hand control system prototype, which is based on the EMG-envelope detection, was implemented to mimic the movement of the human hand. Inside both system prototypes, the CMOS-fabricated ROIC was included and verified to work as their common ExG readout interface. 

## Figures and Tables

**Table 1:** Performance summary and comparison with recent works.

Parameter	This Work	[7]	[12]	[13]
ROIC Application	ECG, EMG, EEG	ECG	ECG	ECG, EEG
Module type	Patch-type ECG module, Portable-type EMG module	N.A.	Ear clip-type ECG module	N.A
Functionality	R-peak detection, EMG envelope detection	Baseline wander tracking	R-peak detection	N.A.
Process	0.18 μm CMOS	N.A.	0.18 μm CMOS	0.18 μm CMOS
Chip Area (mm^2^)	4 mm^2^	N.A.	3.24 mm^2^	24.01 mm^2^
Passband (Hz)	0.6–1500 Hz (programmable)	DC–500 Hz	0.5–22 Hz	1–100 Hz (programmable)
Gain (dB)	31.3–44.8 dB (programmable)	48 dB	47–88 dB (programmable)	47.3–71.9 dB (programmable)
Supply voltage (V)	1.8 V (ROIC) 3 V (Module)	3 V	0.8 V	3.3 V
Power consumption (μW)	37.3 μW (ROIC) 0.2 μW (ADC)	N.A. (ROIC) 160 μW (ADC)	58 nW (ROIC)	12.5 μW (ROIC)
ADC resolution (bit)	12 bit	12 bit	N.A.	12 bit

## Supplementary Materials

The following are available online at http://www.mdpi.com/1424-8220/17/8/1888/s1, Video S1: Comparison experiment results of proposed ECG prototype and commercial ECG holter in stationary and moving states.
